# Epoch length and the physical activity bout analysis: An accelerometry research issue

**DOI:** 10.1186/1756-0500-6-20

**Published:** 2013-01-18

**Authors:** Makoto Ayabe, Hideaki Kumahara, Kazuhiro Morimura, Hiroaki Tanaka

**Affiliations:** 1 Faculty of Health and Sports Science, Fukuoka University, 8-19-1 Nanakuma, Jonan-ku, Fukuoka, 814-0180, Japan; 2 Faculty of Nutritional Sciences, Nakamura Gakuen University, Fukuoka, Japan

**Keywords:** Accelerometer, Pedometer, Pedometry, Methodology, Accuracy, Validity

## Abstract

**Background:**

The purpose of the present investigation was to compare the bouts of daily physical activity (PA) determined by three different accelerometer epoch lengths under free-living conditions.

**Methods:**

One hundred thirty-four adults (50 ± 7 years) wore an accelerometer (Lifecorder) for 7 consecutive days under free-living conditions in order to determine the time spent in physical activity of light intensity (LPA), moderate intensity (MPA), vigorous intensity (VPA), moderate to vigorous intensity (MVPA), and the total physical activity (TPA; sum of LPA, MPA and VPA). Additionally, all PA was divided according to the bout durations (sporadic, >3 min, >5 min, and > 10 min). These indices of PA were analyzed using three different epoch lengths (4 sec, 20 sec and 60 sec) derived from the accelerometer.

**Results:**

The LPA significantly increased in association with increases in the epoch length (48.7 ± 15.9 to 178.7 ± 62.6 min/day, p < 0.05). The amount of sporadic VPA determined by the shortest epoch length (2.9 ± 5.2 min/day) was significantly longer than the two remaining epoch lengths (1.1 ± 2.4 to 0.9 ± 2.5 min/day, p < 0.05). The times of the MVPA bouts lasting longer than 3 minutes determined using the 4-second epoch length (2.6 ± 5.4 to 7.7 ± 10.0 min/day) were significantly shorter than those determined using the other two settings (6.5 ± 10.5 to 13.8 ± 13.8 min/day, p < 0.05). The frequencies of the MVPA bouts lasting longer than 10 minutes determined using the 4-second epoch length (0.2 ± 0.3 bouts/day) were significantly lower than those determined using the other two settings (0.3 ± 0.4 bouts/day, p < 0.05).

**Conclusion:**

The epoch length setting of the accelerometer affects the estimation of the PA bouts under free-living conditions in middle-aged to older adults.

## Background

The American College of Sports Medicine and the American Heart Association recommend that all healthy adults 18 to 65 years of age engage in moderate-intensity aerobic (endurance) physical activity (MPA) or vigorous-intensity aerobic physical activity (VPA) in order to promote and maintain health [1]. Since several consensus statements also recommend engaging in moderate to vigorous intensity physical activity (MVPA) [2,3], spending a longer time performing MVPA is widely accepted to be a goal for a healthy lifestyle. Additionally, moderate-intensity aerobic activity, which is generally equivalent to a brisk walk that noticeably accelerates the heart rate, can count toward the 30 minute minimum by performing bouts of activity lasting 10 minutes or more [1].

Strath et al. [4] showed that bouts of PA lasting ≥ 10 minutes may constitute a more time-efficient strategy to decrease body mass index and waist circumference. In addition, accumulating MVPA in shorter bouts might be a beneficial starting point for individuals to increase their PA levels and decrease their body mass index and waist circumference. Furthermore, sustained volitional activity (i.e., ≥ 10 min in duration) might play an important role for the long-term maintenance of weight loss [5]. These findings indicate that participating in MVPA lasting > 10 min might be more beneficial than accumulating sporadic MVPA.

However, with regard to bouts of MVPA lasting > 10 minutes, no consistent definition has been used to evaluate activity monitor data, and the traditional use of 1 minute recording intervals seems to be the *de facto* bout definition [6]. Accelerometers function by integrating a filtered digitized acceleration signal over a user-specified time interval, commonly referred to as an epoch [7]. The usual accelerometer stored magnitude of accelerations at fixed recording intervals (1 sec, 4 sec, 15 sec, or 60 sec or longer) is called an “epoch” [8]. At the end of each epoch, the some index of physical activity is calculated; this process is repeated until data collection is completed [8,9]. Recent studies examined the appropriate epoch length to estimate the sporadic MVPA [7,8,10], and the use of a shorter time sampling interval might reduce the misclassification errors of PA estimates [8]. However, there have been no studies that have directly compared the bouts of PA determined using different epoch lengths under free-living conditions. Since most walking bouts last 30 sec or less [11,12], we hypothesized that a shorter epoch length may be more suitable to reflect the “real” bout duration under free-living conditions.

Therefore, the purpose of the present investigation was to examine the effects of the epoch length lasting from 4 seconds to 1 minute on the evaluation of bouts of PA lasting longer than 3, 5 and 10 minutes under free-living conditions in middle- to older-aged adults.

## Methods

### Subjects

One hundred and thirty-four adults, aged from 32 to 72 yr., participated in the present investigation. All volunteers were recruited from Fukuoka city (Fukuoka, Japan) by the newspaper and web site. All volunteers living independently and were free of debilitating chronic diseases. Furthermore, none of the participants habitually participated in any sports activities. The characteristics of the participants are shown in Table 1 (Table 1). After an explanation of the study requirements, each subject read and signed a consent form. The ethics committee of Fukuoka University approved all procedures used in the present investigation. All procedures related to the measurements were performed between March and April.

**Table 1:** Characteristics of participants

	**All**	**Female**	**Male**
N	134	42	92
Age (years)	50 ± 7	50 ± 7	50 ± 8
Height (cm)	165.0 ± 8.1	156.2 ± 4.9	169.1 ± 5.8*
Body weight (kg)	66.7 ± 12.4	54.7 ± 8.4	72.3 ± 9.8*
Body mass index (kg/m^2^)	23.3 ± 6.9	28.5 ± 6.9	21.0 ± 5.5*
Waist circumference	88.2 ± 8.8	84.3 ± 9.7	90.0 ± 7.8*

Data are expressed as the means with the standard deviation (mean ± SD).

*Significantly different between males and females (p < 0.01).

### Physical activity assessments

During the course of the present investigation, all participants wore a pedometer with a uni-axial accelerometer (Lifecorder Ex 4-sec version, Kenz, Nagoya, Japan; LC) under free-living conditions. The LC is a small and lightweight (60 mm × 46 mm × 26 mm, 42 g) device that is equipped with a uni-axial piezo-electronic accelerometer that samples vertical acceleration ranging between 0.06 G and 1.94 G at 32 Hz. Based on the magnitude and frequency of accelerations, the LC determines the level of movement intensity every 4 seconds on a scale of 1 (minimal intensity of movement) to 9 (maximal intensity of movement). As shown in a previous investigation [9], the intensity levels closely approximated the metabolic equivalents (METs) of the activities being performed. Previous investigations have demonstrated that the LC is suitable for research purposes [9,13,14]. The LC has the potential limitation, similar to other types of activity monitors, in that they too often remain vulnerable to problems from external vibrations and unmeasured sources of energy expenditure such as hill climbing and isometric activity in daily life [15]. After reading the instructions regarding the general care of these activity monitors, the participants wore the LC for 10 days continuously, except while sleeping or bathing. We used seven continuous days (the 4th day to the 10th day, five weekdays and two weekend days) for data the analysis because the 1st to the 10th days included non-wearing time and the 2nd day was used to familiarize the subjects with the LC. All subjects documented when they removed the LC die for personal reasons. The LC was placed on the left anterior mid-line of the thigh on the waist band of the participant’s clothing. The present investigation placed the LC on the left side in order to prevent the LC from being dropped while the subjects inserted or removed their belts. The LC was sealed, so that the participants could not gain access to the PA measurements during the experimental period. After the data collection period was completed, the participants returned the LC by mail to the investigators so that the stored data could be uploaded to a personal computer for the analysis. To be included in the analysis, each participant had to have at least seven days of sufficiently wearing the device (>10 hours) [7].

### Accelerometer’s data analysis

All accelerometer’s out puts were averaged over 7 days, and weekly values were defined as individuals represented. Since the present investigation was focused on the accelerometer’s algorithms, the inter individual variability, such as the day-to-day variability or hourly variability, were not evaluated according to the previous validation studies [8,14]. In the present investigation, the epoch length was also set at 20 sec and 60 sec, in addition to the usual 4 sec setting. The reasons why we chose these three epoch lengths are as follows: first, the 4 sec and 60 sec epoch lengths were used in previous studies [6,16,17]. Additionally, as the LC detects the intensity of PA every 4 sec, the epoch length had to be selected from a multiple of 4 sec, and the epoch length must be a common divisor of the bout duration (3 min, 5 min and 10 min). Therefore, we chose 20 sec as the third epoch length.

**Figure 1:**
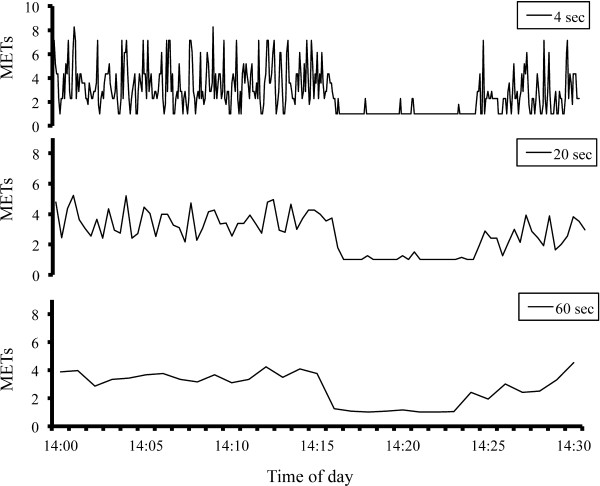
**Accelerometer outputs as determined using three different epoch lengths.** Each illustration shows the MET values from 14:00 to 14:30 in one of the participants. The upper, middle and lower illustrations indicate the MET values determined by the epoch lengths of 4, 20 and 60 seconds, respectively. For the epoch length of 4 seconds, no PA bouts lasting > 10 minutes were detected. For the epoch length of 20 seconds, one PA bout lasting > 10 minutes was detected (14:03 to 14:15). For the epoch length of 60 seconds, one MVPA bout lasting > 10 minutes was detected (14:03 to 14:15).

The time spent in light intensity PA (LPA), moderate intensity PA (MPA), vigorous intensity PA (VPA), and moderate + vigorous intensity PA (MVPA) was determined. Furthermore, the sum of the time for LPA, MPA, and VPA was defined as the total PA (TPA). Thereafter, based on the time series data of the three epoch lengths, the TPA and MVPA were divided according to the bout durations (Sporadic, < 3 min, < 5 min, and < 10 min, respectively). We defined a “bout” as PA that was maintained completely at an intensity level of 1 or higher for at least 3 minutes, 5 minutes or 10 minutes (TPA3, TPA5, TPA10, and TPA3, TPA5, TPA10, respectively). Similarly, the daily time spent for the MVPA lasting at least 3 minutes, 5 minutes and 10 minutes were assessed (MVPA3, MVPA5, and MVPA10, respectively). The reason for using these durations was as follows: First, only the 3 min bouts of PA had a significant impact on the post-prandial blood lipids and metabolic syndrome in previous studies [16,18,19]. Furthermore, the bouts of PA lasting longer than 5 min comprised just 0.14 percent of all walking bouts under free-living conditions [11,12]. Finally, the PA bouts lasting longer than 10 min have been widely recommended by consensus statements [1] (Figure 1).

### Statistics

**Figure 2:**
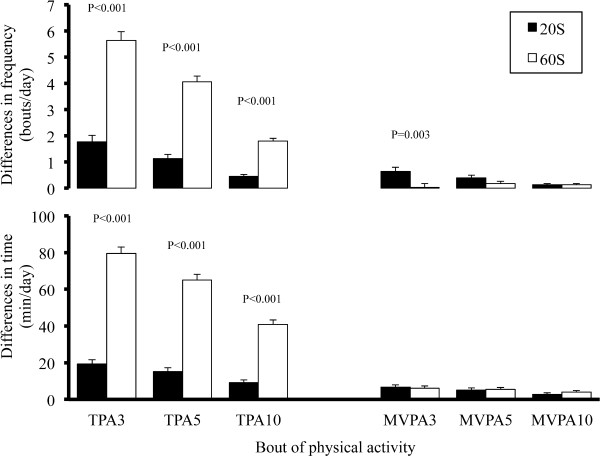
**Differences in the time and frequency of physical activity bouts compared with those determined using a 4 sec epoch length.** The Y-axis shows the differences between the time or the frequency spent on the physical activity bout determined by the 4 sec epoch length and that by the 20 sec or 60 sec epoch length, respectively. TPA3, TPA5, TPA10; total physical activity lasting longer than 3 min, 5 min and 10 min. MVPA3, MVPA5, MVPA10; moderate to vigorous intensity physical activity lasting longer than 3 min, 5 min and 10 min.

The data were expressed as the means with standard deviations. The potential gender-related differences in the obtained data were analyzed by the unpaired *t*-test. A one-way ANOVA and Scheffe’s test were used to compare the PA levels for the 3 different epoch lengths. The magnitude of the differences between the time and frequency of PA for the 4 sec epoch length compared to those of the other two epoch lengths was analyzed by paired *t*-tests. Statistical significance was set at P < 0.05. All statistical analyses were performed using the StatView software program (version 5.0.1, SAS Institute, Cary, NC, USA) (Figure 2).

## Results

The height, body weight, body mass index, and waist circumference were significantly different between males and females (p < 0.01, Table 1). As none of the accelerometer outputs significantly differed between genders, the PA data are shown as the combined results for males and females (Tables 2 and 3). Table 2 shows the time spent in sporadic PA as determined by the three different accelerometer epoch lengths. The LPA significantly increased with the increase in epoch length (p < 0.05). The amount of sporadic VPA determined by the shortest epoch length (4 sec) was significantly longer compared with the two remaining epoch lengths (p < 0.05). Table 3 shows the time and frequency for the PA bouts as determined using the three different accelerometer epoch lengths. Regardless of the intensities and bout durations, the frequency and the time for the PA bouts determined using the 4 sec epoch length were significantly shorter compared with the other two settings (p < 0.05). Additionally, the frequency and the time for TPA determined using the 20 sec epoch length were significantly smaller compared with that determined using the 60 sec epoch length (p < 0.05).

**Table 2:** Accelerometer’s epoch length and the time spent in sporadic physical activity

	**Epoch length**		
	**4 sec**	**20 sec**	**60 sec**
LPA (min/day)	48.7 ± 15.9	104.2 ± 36.1^**^	178.7 ± 62.6^**, ‡^
MPA (min/day)	30.0 ± 14.9	33.2 ± 16.2	28.5 ± 15.9^*,†^
VPA (min/day)	2.9 ± 5.2	1.1 ± 2.4^**^	0.9 ± 2.5^**^
MVPA (min/day)	32.9 ± 16.5	34.3 ± 17.1	29.4 ± 16.9^*,†^

Data are expressed as the means with the standard deviation (mean ± SD). LPA; light intensity physical activity, MPA; moderate intensity physical activity, VPA; vigorous intensity physical activity, MVPA; moderate to vigorous intensity physical activity.

^*, **^Significantly different compared with 4 sec (^*^p < 0.05, ^**^p < 0.01). ^†, ‡^Significantly different compared with 20 sec (^†^p < 0.05, ^‡^p < 0.01).

**Table 3:** Frequency and time in bouts of physical activity by 3 different accelerometer’s epoch lengths

		**Epoch length**		
		**4 sec**	**20 sec**	**60 sec**
Frequency (bouts/day)	TPA3	3.3 ± 1.8	5.1 ± 2.0^**^	9.0 ± 3.3^**,‡^
	TPA5	1.5 ± 1.1	2.7 ± 1.4^**^	5.6 ± 2.1^**, ‡^
	TPA10	0.4 ± 0.5	0.8 ± 0.8^**^	2.2 ± 1.1^**, ‡^
	MVPA3	1.1 ± 1.3	1.8 ± 1.3^**^	1.2 ± 0.9^**, ‡^
	MVPA5	0.5 ± 0.8	0.9 ± 0.8^**^	0.7 ± 0.7^**^
	MVPA10	0.2 ± 0.3	0.3 ± 0.4^**^	0.3 ± 0.4^**^
Time (min/day)	TPA3	22.0 ± 15.1	41.3 ± 21.6^**^	101.8 ± 37.6^**, ‡^
	TPA5	14.8 ± 13.2	30.0 ± 20.3^**^	80.2 ± 33.2^**, ‡^
	TPA10	6.9 ± 9.9	15.9 ± 17.0^**^	47.7 ± 27.8^**, ‡^
	MVPA3	7.7 ± 10.0	14.3 ± 12.8^**^	13.8 ± 13.8^**^
	MVPA5	5.2 ± 8.1	10.3 ± 11.2^**^	10.6 ± 12.8^**^
	MVPA10	2.6 ± 5.4	5.4 ± 8.8^**^	6.5 ± 10.5^**^

Data are expressed as the means with the standard deviation (mean ± SD). TPA3, TPA5, and TPA10; Bouts of physical activity lasting longer than 3 min, 5 min and 10 min. MVPA3, MVPA5, and MVPA10; Bouts of moderate to vigorous intensity physical activity lasting longer than 3 min, 5 min and 10 min.

^**^Significantly different compared with 4 sec (^**^p < 0.01). ^†, ‡^Significantly different compared with 20 sec (^†^p < 0.05, ^‡^p < 0.01).

Figure 2 shows the differences in the times and frequencies of the bouts of TPA and MVPA between those determined using the 4-second epoch length and those determined using the 20- and 60-second epoch lengths. The magnitudes of the differences in the times and frequencies of the bouts of TPA determined using the 60-second epoch length were significantly larger than those determined using the 20-second epoch length (p <0.001). In contrast, the magnitudes of the differences in the times and frequencies of the bouts of MVPA did not differ significantly, except for the time of the MVPA_3M_.

## Discussion

The present investigation is, to the best of our knowledge, the first study to examine the role of an accelerometer’s epoch length (4 sec, 20 sec and 60 sec) in the evaluation of PA bouts with regard to accelerometer-based PA assessment in middle- to older-aged individuals. Main finding of the present investigation is that, regardless of the intensity and bout duration categories, the frequency and time for the PA bouts were significantly lower when the shortest epoch length (4 sec) was used compared with the longer epoch lengths (20 sec and 60 sec). Furthermore, when the analysis was limited to the TPA, the frequency and time for the PA bouts were significantly lower as the epoch length increased. Although previous investigation examined the epoch length on the “sporadic MVPA estimation” [7,8,10], the present investigation firstly showed that the epoch length affects the “MVPA bouts estimation”. These results indicate that the accelerometer’s epoch length affects the estimation of the PA bouts under free-living conditions in middle-aged to older adults. The 20- and 60-second epoch lengths resulted in larger amounts of MVPA bouts compared with the 4-second epoch length. Thus, caution should be exercised regarding the accelerometer’s data setting when discussing the PA bouts reported in previous publications. Furthermore, the best epoch length to estimate MVPA bouts remains unclear.

One of the original findings of the present investigation is that, with regard to the accelerometer-based PA assessment, a shorter epoch length results in smaller amounts of PA bouts compared with the longer epoch lengths under free-living conditions in middle aged to older adults. Although several previous investigations have examined the effects of an accelerometer’s epoch length on the estimation of sporadic MVPA [6,8,20], the present investigation provides the first demonstration that an accelerometer’s epoch length affects the estimation of the frequency and time of PA bouts. A previous study showed that a shorter epoch length was more suitable for estimating the time spent in sporadic MVPA, where MVPA were significantly higher and light-intensity activity was significantly lower when presented as 10 sec epochs compared with that using a 60 sec epoch length [8]. Similar results are also found in adolescents and children where a significant epoch effect was seen for time spent in VPA, LPA, and rest in the child and adolescent samples and MVPA in the child sample [10]. These findings are consistent with the results of the present investigation, where the daily time spent in sporadic MPA and VPA based on the shortest epoch length (4 sec) were significantly longer than that based on the longer epoch lengths (Table 2). However, with regard to the PA bouts, it is of interest that the relationships between the accelerometer’s epoch length and the amount of PA were inverted (Table 3). The frequency and time for the PA bouts were significantly smaller for the shorter epoch length setting compared with the longer epoch length settings. The mean daily time for MVPA_10_ under the shortest epoch length setting was less than half of that under the longer epoch length settings.

The main explanation for the differences in the amount of PA among the three different epoch lengths is considered to be due to two different factors. First, the bout duration of daily PA is very short under free-living conditions [12,16,17]. Orendurff et al. previously indicated that 60% of all walking bouts lasted less than 30 seconds, and 81% of all walking bouts lasted 1 minute or less, and 2 minute walking bouts represented just 1% of all walking bouts [12]. Second, the shorter epoch has a higher sensitivity to detect the changes in the human PA, such as the transition to PA from rest, and/or the switch from walking to jogging, etc., compared with the longer epoch length. Under the 1 min epoch length, for example, if people walk for five seconds then rest for 55 seconds, this 1 min would be categorized as LPA or MVPA rather than a resting period. Similarly, if people take a short break during continuous jogging, this minute would be categorized as MPA rather than VPA, regardless of the presence or absence of the break. In other words, with regard to the 60 sec epoch length, the intensity of PA is less likely to result in a classification of inactivity or VPA, because the various types of PA, including the inactivity, are averaged over 1 min periods. As a result, the accelerometer might detect a larger number of PA bouts. In contrast, a shorter epoch length can more sensitively detect the changes in the type and intensity of PA, as well as the resting period (brief interruptions, etc.). Thereafter, the PA bouts were significantly smaller for the shorter epoch length compared with the longer epoch length.

There is large variability in MVPA bouts lasting longer than 10 minutes. In the present investigation, the mean MVPA lasting > 10 minutes was 2.6 ± 5.4 min day^-1^ under the 4-second epoch length. Similarly, the time for MVPA bouts was 2.0 min day in the US NHANES Study [21], 0.4 to 0.6 min day^-1^ in British, Italian and French volunteers [22] and 0.0 to 5.2 min day^-1^ in a Japanese study [23]. In contrast, some investigators have reported much longer MVPA bouts of > 30 min day^-1^ in overweight and obese individuals [24], 19.2 ± 18.6 min day^-1^ in overweight participants and 25.8 ± 23.4 min day^-1^ in normal-weight individuals [5]. The differences in the amounts of MVPA bouts seem to exceed the acceptable range and are caused not only by the epoch length, including human-behavioral elements, but also by the data analysis process. In other words, the procedures used to assess and quantify the PA bouts must evolve in order to determine how to manage brief interruptions (i.e. breaks of < 1 minute) of PA. In the present investigation, all MVPA bouts satisfied the inclusion criteria, where the intensity of PA was above 3 METs for that lasting at least 10 minutes. In contrast, some investigators have attempted to improve the sensitivity of detecting MVPA bouts. For example, to allow for the brief periods of rest common during certain activities, i.e. pausing for a water break while playing basketball, the criteria used to define a bout requires a running average of 70% of the counts to be above the cut-off point [21]. Similarly, interruptions of 1 or 2 minutes below 3 METs are allowed during consecutive MVPA lasting > 10 minutes to define bouts of MVPA [25]. We cannot conclusively define the best epoch length to estimate PA bouts because we were unable to compare the accelerometer’s outputs with the “gold standard method for assessing PA bouts.” Because it is difficult to assess the bout duration of PA under free-living conditions, using video tape recordings and/or direct observation is the gold standard method for assessing PA bouts in experimental studies.

However, the previous and present investigations apparently indicate that a shorter epoch length may be superior to estimate the PA bouts compared to a longer epoch length [8,26]. Furthermore, recent publications showed that, although PA bouts are relatively rare under free-living conditions, the MVPA lasting > 1 min was significantly associated with the blood lipid status and age in previous studies [16,17]. In contrast, with regard to the 1 min epoch length, it is doubtful whether it can evaluate “real” PA bouts under free-living conditions, due to the lack of accurate treatment of the interruptions in activity. Further investigations will be required to determine how to divide the continuous PA with interruptions and the intermittent PA, how to assess the activity level during these short interruptions, and what length of interruptions can be accepted, etc. However, a recent publication, which summarized accelerometer-based research, showed that the longer epoch duration (1 minute) is the most commonly used epoch length [6]. In addition, the time for MVPA bouts lasting > 10 minutes based on the 60-second epoch length is also significantly associated with health outcomes using this length [4,5]. Additionally, using a longer epoch length can reduce the data to be saved, so the results using a longer epoch length would be superior with regard to data treatment, and would allow for a longer monitoring period by saving the data storage capacity. Therefore, each epoch length has both advantages and disadvantages. Future research should first develop a method to quantify the PA bouts on the basis of a detailed analysis, thereafter, the contribution of the different PA bouts to the health outcomes should be examined based on the quantitative assessment of the PA bouts based on the most valid epoch length.

There are several limitations to the present investigation that should be considered when interpreting the results. First, the type (uni-axial) and position (waist) of the accelerometer used in the present investigation does not allow for the collection of upper body activity, and thus may underestimate the total PA. Additionally, as the LC has an original algorithm for PA intensity classification, the results of the present investigation should be confirmed using other activity monitors. Second, the participants evaluated in the present investigation were primarily middle-aged, non-active males and females. Furthermore, the participants all lived in urban areas, and buses and trains were their primary means of transportation. Thus, it is limited to generalize the present investigation to the heterogeneous individuals, therefore, the results of the present investigation should be confirmed in active individuals and heterogeneous volunteers, including young children.

## Conclusion

In summary, the present investigation was the first to examine the role of the accelerometer’s epoch length on the evaluation of the PA bouts with regard to the accelerometer-based PA assessment. The frequency and time for the PA bouts were significantly lower as the epoch length increased. These results indicate that the accelerometer’s epoch length affects the estimation of the PA bouts under free-living conditions in middle-aged to older adults. Thus, caution should be exercised when selecting the accelerometer’s data setting, and when discussing the results of previous studies about the PA bouts. The best epoch length to estimate MVPA bouts remains unclear, and will need to be assessed in future studies. In order to generalize the results of the present investigation, future studies should examine the effects of the epoch length in heterogeneous volunteers using several types of activity monitors.

## Competing interests

None of the authors have any professional relationship with companies or manufactures that will benefit from the results of the present study.

## Authors’ contributions

Planning: MA, HT, Conducting: MA, HK, KM, Data treatment: MA, KM, Reporting: MA, HK, HT. All authors read and approved the final manuscript.
